# Early Thalamic Injury After Resuscitation From Severe Asphyxial Cardiac Arrest in Developing Rats

**DOI:** 10.3389/fcell.2021.737319

**Published:** 2021-12-07

**Authors:** Hoai T. Ton, Katherine Raffensperger, Michael Shoykhet

**Affiliations:** Center for Neuroscience Research, Children’s National Hospital, Children’s Research Institute, Washington, DC, United States

**Keywords:** cardiac arrest, thalamic reticular nucleus, GABA-ergic interneuron, microglia, neuronal degeneration, hypoxia, ischemia, reperfusion

## Abstract

Children who survive cardiac arrest often develop debilitating sensorimotor and cognitive deficits. In animal models of cardiac arrest, delayed neuronal death in the hippocampal CA1 region has served as a fruitful paradigm for investigating mechanisms of injury and neuroprotection. Cardiac arrest in humans, however, is more prolonged than in most experimental models. Consequently, neurologic deficits in cardiac arrest survivors arise from injury not solely to CA1 but to multiple vulnerable brain structures. Here, we develop a rat model of prolonged pediatric asphyxial cardiac arrest and resuscitation, which better approximates arrest characteristics and injury severity in children. Using this model, we characterize features of microglial activation and neuronal degeneration in the thalamus 24 h after resuscitation from 11 and 12 min long cardiac arrest. In addition, we test the effect of mild hypothermia to 34°C for 8 h after 12.5 min of arrest. Microglial activation and neuronal degeneration are most prominent in the thalamic Reticular Nucleus (nRT). The severity of injury increases with increasing arrest duration, leading to frank loss of nRT neurons at longer arrest times. Hypothermia does not prevent nRT injury. Interestingly, injury occurs selectively in intermediate and posterior nRT segments while sparing the anterior segment. Since all nRT segments consist exclusively of GABA-ergic neurons, we asked if GABA-ergic neurons in general are more susceptible to hypoxic-ischemic injury. Surprisingly, cortical GABA-ergic neurons, like their counterparts in the anterior nRT segment, do not degenerate in this model. Hence, we propose that GABA-ergic identity alone is not sufficient to explain selective vulnerability of intermediate and posterior nRT neurons to hypoxic-ischemic injury after cardiac arrest and resuscitation. Our current findings align the animal model of pediatric cardiac arrest with human data and suggest novel mechanisms of selective vulnerability to hypoxic-ischemic injury among thalamic GABA-ergic neurons.

## Introduction

Cardiac arrest affects 12–18,000 children each year in the United States alone (Donoghue et al., 2005). It contributes to ∼30% of all pediatric deaths (Fink et al., 2016) and is a leading cause of brain injury in children (Graves et al., 1997; Maryniak et al., 2008; Ichord et al., 2018). No current treatments are available. Multiple treatment approaches have shown benefit in animal models of pediatric cardiac arrest, yet none has been translated into clinical practice. Even therapeutic hypothermia, with its broad molecular and physiologic impact, has failed to improve neurologic outcomes in pediatric cardiac arrest (Moler et al., 2015; Moler et al., 2017). A different approach to bridge the bench-bedside divide is needed.

One of the difficulties in translating findings in animal models to humans is the disparity in the severity of injury. Traditionally, animal models of cardiac arrest have focused on relatively mild injury with targeted survival >90%. Yet, in humans, cardiac arrest survival is <10% (Fink et al., 2016; Yan et al., 2020). Arrest times in animal models are short (7–9 min) compared to those observed in humans (∼11 ± 2 min) (Young et al., 2004; Herlitz et al., 2005; Herlitz et al., 2007; Goto et al., 2014; Tijssen et al., 2015). Long-term behavioral deficits in animal cardiac arrest survivors are relatively mild, comprised of learning and memory impairments on common laboratory tasks (e.g. Morris water maze) (Neumann et al., 2013). Yet, 50% of human cardiac arrest survivors experience severe neurologic deficits such as paralysis, spasticity (Scheibe et al., 2020), seizures and disorders of consciousness (Lim et al., 2004; Maryniak et al., 2008; Moler et al., 2015). Thus, in order to effectively test therapeutic strategies in pre-clinical cardiac arrest models, injury severity in animals must better approximate severity observed in humans.

An additional confounding factor has been historical focus on the delayed death of hippocampal CA1 neurons (Kirino, 2000). While this focus yielded several candidate mechanisms of cellular injury after hypoxia-ischemia-reperfusion (Neumann et al., 2013), therapies targeting these mechanisms have rarely been assessed in other neuronal populations susceptible to injury. Indeed, the very nature of neuronal populations affected by the more severe insult is less well characterized in animal models (Hogler et al., 2010). In humans, on the other hand, there is substantial evidence from MRI studies that deeper brain structures such as the basal ganglia and the thalamus are susceptible to hypoxic-ischemic injury (Fink et al., 2020; Vanden Berghe et al., 2020).

Thalamic injury in particular may have a profound effect on post-arrest recovery and neurologic function. Conscious perception of all senses, except olfaction, requires processing and relay of information from the thalamic sensory nuclei to the cerebral cortex. Decision making requires intact corticothalamic loops involving the mediodorsal thalamus (de Kloet et al., 2021). Attention (Wells et al., 2016) and sleep (Halassa et al., 2011) require an intact thalamic reticular nucleus. Even in the absence of overt cortical injury, isolated thalamic injury, as seen in necrotizing thalamic encephalitis (Wong et al., 2006) and in thalamic strokes (Fritsch et al., 2021), leads to dismal neurologic outcomes. We have previously demonstrated evidence of long-term thalamocortical circuit dysfunction after a relatively mild cardiac arrest during development (Shoykhet et al., 2012; Aravamuthan and Shoykhet, 2015; Shoykhet and Middleton, 2016; Middleton et al., 2017). Hence, a clear need exists for understanding which populations of thalamic cells are most susceptible to injury, the specific cellular mechanisms involved in injury to these populations, and the functional consequences of such injury.

Here, we develop a model of prolonged pediatric asphyxial cardiac arrest in developing rats with ischemia times and post-arrest physiologic disturbances matching those observed in children. We then characterize microglial activation and neuronal degeneration in the thalamus 24 h after resuscitation as a necessary first step towards understanding how injury to thalamic microglial and neuronal circuits contributes to post-arrest neurologic deficits.

## Methods

### Animals

All experimental procedures involving animals were approved by the Institutional Animal Care and Use Committee at Washington University School of Medicine. Long Evans rats (Envigo, IL) at postnatal days (PND) 17–19 (day of birth = PND 0; *n* = 14) were used in the experiments. Rat brain development at this age corresponds roughly with that of a 2–4 years/old child (Semple et al., 2013), allowing us to model pediatric cardiac arrest outside of the neonatal period but still within the time window for ongoing brain maturation. Rats were housed with their mother in a temperature- and humidity-controlled environment with free access to water and food. The animals underwent 11 min (*n* = 3/3/3 arrested/resuscitated/survived 24 h), 12 min (*n* = 4/4/3 arrested/resuscitated/survived 24 h), 12.5-min + hypothermia (*n* = 6/5/5 arrested/resuscitated/survived 24 h) asphyxial cardiac arrest or sham (*n* = 3/3 sham surgery/survived 24 h) intervention. The animals were randomized by means of a sealed envelope. Histochemical and immunohistochemical experiments were performed on these groups 24 h after injury or sham treatment. The goal of this study is to characterize early neuronal degeneration as opposed to delayed neuronal death observed 3–7 days after injury (Tang et al., 2010; Shoykhet et al., 2012) This time point was chosen based on prior experiments as the earliest at which neuronal degeneration may be observed (Shoykhet et al., 2012). We used both male and female rats. The experimenter was blind to the injury status of the rats during image and statistical analyses.

### Cardiac Arrest and Resuscitation

We further extended a previously described rat model of pediatric asphyxial cardiac arrest to produce severe injury comparable to that observed in children (Fink et al., 2004; Shoykhet et al., 2012). The ischemia time was lengthened to a maximum of 12.5 min. Prolonged cardiac arrest followed by resuscitation in this model results in ∼ 4–6 h of cardiovascular dysfunction, ∼8–12 h of coma and signs of spasticity observed as early as 12 h post-injury (Aravamuthan and Shoykhet, 2015). Due to injury severity, the resuscitated animals required up to 12 h of post-arrest critical care including invasive mechanical ventilation, continuous fluid and inotropic support, and temperature regulation with a homeostatic heating blanket. Sustained intensive care improved the 24 h survival rate from <50% in preliminary experiments to ∼85% (11/13) in this series.

**FIGURE 1:**
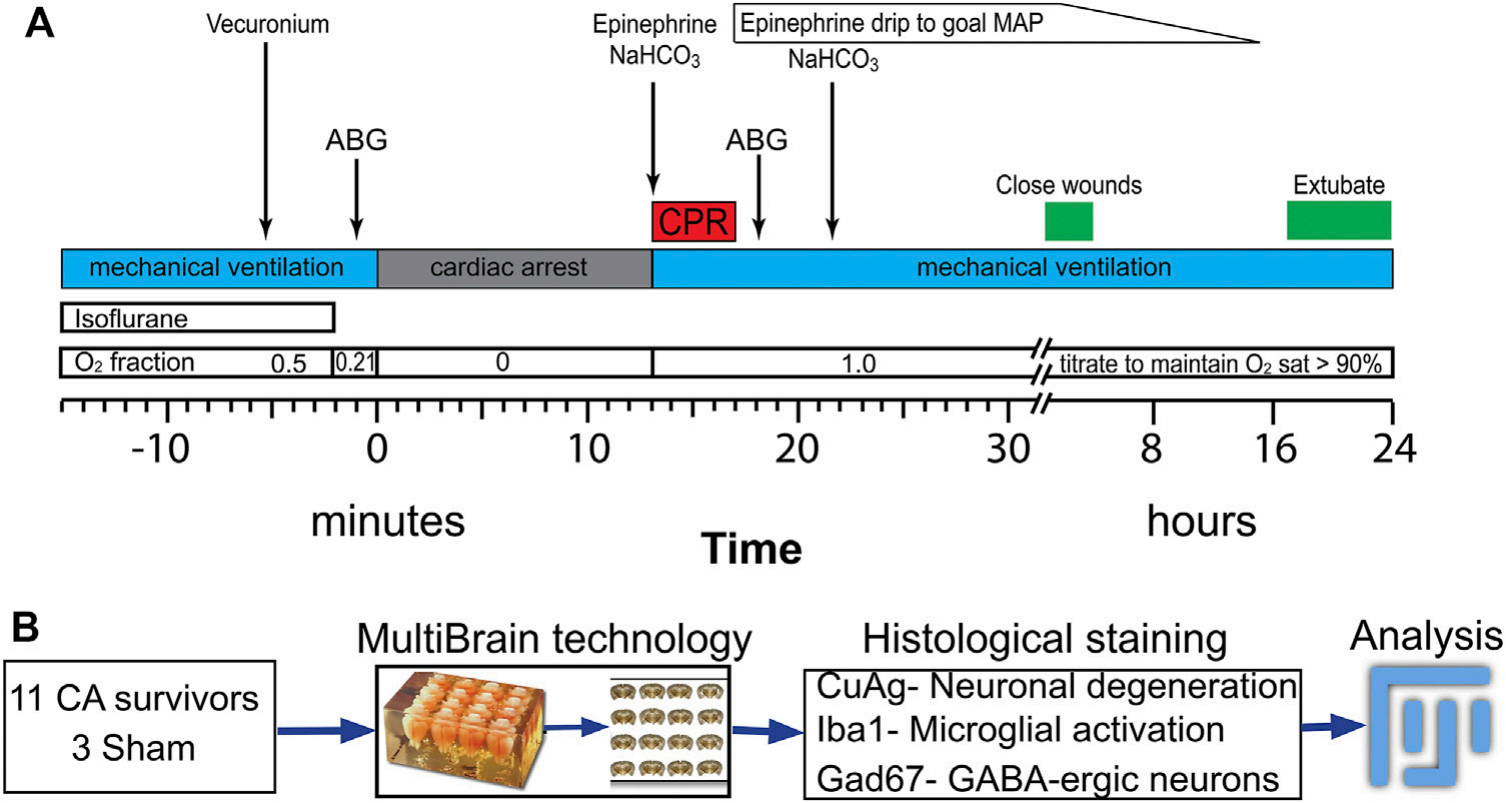
Procedure for asphyxia cardiac arrest and resuscitation experiment following by tissue processing for histological study **(A)** Timeline and procedure for anesthetic washout, asphyxia, PCR and post-ROSC periods. **(B)** Experimental procedure for histological staining and analyzing after 24 h after insults. ABP: artery blood pressure; ECG: electrocardiogram; CA: cardiac arrest; CPR: cardiopulmonary resuscitation; ROSC: return of spontaneous circulation; MAP: mean arterial pressure.

Figure 1 shows the general workflow for asphyxial cardiac arrest and resuscitation followed by tissue processing. Briefly, PND 17–19 rats were anesthetized with isoflurane in 50/50 O_2_/N_2_ mixture, endotracheally intubated and maintained with pressure-controlled, time-cycled mechanical ventilation (Positive End Expiratory Pressure (PEEP) = 5, Peak Inspiratory Pressure (PIP) = 15–18, Rate 70–90 /min, I:E ratio 1:2). Femoral arterial and external jugular venous cannulas were placed for blood pressure monitoring and drug administration, respectively. Needle electrocardiogram (ECG) and electroencephalogram (EEG) electrodes were inserted subcutaneously. Respiratory parameters, arterial blood pressure (ABP), pulse oximetry, ECG, EEG, and end-tidal CO_2_, were continuously monitored and recorded (PowerLab, ADInstruments, CO). Rectal temperature was maintained at 37.0°C with a homeostatic heating blanket (SurgiSuite, Kent Scientific Corporation, CT). Mechanical ventilation parameters were titrated to maintain normal oxygenation and ventilation as evidenced by arterial blood gas (ABG) measurements before arrest and 10 min after resuscitation (ABL90 Flex, Radiometer). After completion of all surgical procedures, vecuronium (2 mg/kg ip, Teva Pharmaceutical) was used to establish neuromuscular blockade. Two minutes prior to cessation of mechanical ventilation, the ventilator gas mixture was changed to room air (FiO_2_ = 0.21) to wash out isoflurane and excess oxygen. This anesthetic wash-out period minimizes the confounding effects of isoflurane on neuronal injury and resuscitation. During the wash-out period, EEG was monitored to prevent awakening. One minute into the washout period (i.e. 1 minute prior to arrest), a pre-arrest ABG was obtained to verify adequacy of oxygenation and ventilation. Mechanical ventilation was then stopped to induce asphyxial cardiac arrest. When starting with room air (arterial pO_2_ 60–100 mm Hg), asystole with electro-mechanical dissociation (Pulseless Electrical Activity, PEA) occurs within 40–60 s. At the end of the predetermined period of asphyxia, the rats were resuscitated with mechanical ventilation (FiO2 = 1, PEEP = 5, PIP and rate increased 20% over baseline) and manual chest compressions (∼300 /min). Chest compressions were titrated in real time to target a diastolic blood pressure >20 mm Hg. Epinephrine (0.01 mg/kg iv, Par Pharmaceutical) and NaHCO_3_ (1 mEq/kg 4) were administered 1 min into the resuscitation and repeated once if no return of spontaneous circulation (ROSC) occurred in the first 2 min of resuscitation. Sham rats underwent all procedures except arrest and resuscitation.

### Post-Arrest Intensive Care

Ten minutes after ROSC, a post-arrest ABG was obtained. Mechanical ventilation was adjusted as needed and an additional dose of NaHCO_3_ was given to correct the acidosis. ABP was monitored continuously, and an epinephrine infusion was initiated to maintain MAP >80% of baseline. Epinephrine infusion (0.1–0.8 μg/kg/min) was required for 2–4 h post-arrest to maintain adequate hemodynamics. Rectal temperature was maintained at 37.0°C for normothermic rats and at 34.0°C for rats treated with mild hypothermia. Temperature control was continued until extubation ∼12 h post-arrest. Neurologic status of the animal was monitored via EEG and observation of spontaneous respirations as well as response to gentle whisker stimulation. Post-arrest EEG demonstrated progression from electrical silence to burst suppression to more organized rhythms. Emergence from burst suppression on EEG was followed by initiation of spontaneous breaths and whisker twitch to gentle air puffs. When spontaneous breaths became more frequent, mechanical ventilation was weaned to an assisted pressure-support mode with a back-up rate of 60 min. The back-up rate was weaned gradually until the animal could maintain spontaneous ventilation, often accompanied by frequent yawning. The rat was then extubated to a nose cone supplying blow-by O_2_. Throughout the weaning process, pulse oximetry was monitored continuously to maintain peripheral hemoglobin O_2_ saturations >92%.

### Histochemistry and Immunohistochemistry

Twenty-four hours after injury or sham operation, rats were deeply anesthetized with 5% isoflurane in 100% oxygen and perfused transcardially with cold PBS followed by 4% paraformaldehyde solution. The brains were left *in situ* immersed in fixative for an additional 24 h to minimize artifact (Garman, 1990) and then removed and post-fixed for 48 h. The entire cohort of brains was then processed simultaneously using MultiBrain technology (NeuroScience Associates, Knoxville, TN). Brains were sectioned at 40 µm and stained for neuronal degeneration using amino cupric silver (DeOlmos and Ingram, 1971; Switzer, 2000), for microglia using anti-Iba1antibody (FUJIFILM Wako Pure Chemical Corporation, Cat# 019–19,741, diluted 1:12,000), and for inhibitory neurons using anti-Gad67 antibody (AbCam, ab26116, diluted 1:30,000). All antibodies were visualized with Ni(II) diaminobenzidine. Detailed staining protocol from NeuroScience Associates, Inc. is included in Supplementary Material.

### Image Acquisition and Statistics

The images were obtained with a Microlucida system (MicroBrightField) with an Axioskop microscope driven stage and an AxioCam MRc camera (Zeiss Microscopy). All imaging was performed in batches where a set of sections representing all groups were processed simultaneously. The stained areas were quantified in FIJI (Schindelin et al., 2012) with uniform scale across all images using The Rat Brain Atlas (Paxinos and Watson, 2007) to visually guide localization of the regions of interest (ROI). Data are presented as individual values with median and interquartile range. One-way ANOVA, nested one-way ANOVA with Dunnett’s multiple comparisons (in Figures 3D–D) and nested *t*-test (in Figures 6C and Figure 8C) were used as appropriate for statistical analyses. The brain regions with respect to bregma that were examined in all the groups are diagrammed in Figures 3C, Figure 4A, Figure 5 and Figure 8B.

## Results

### Arrest Characteristics

All resuscitated rats required mechanical ventilation for 8–12 h and epinephrine infusion (max dose 0.8 μg/kg/min) for 2–4 h after resuscitation. Post-arrest whole blood lactate levels obtained 10 min after resuscitation increased with increasing arrest duration (in mg/dL, Sham 1.8 ± 0.6, 11 min 8.0 ± 0.6, 12 min 9.9 ± 1.9, 12.5 min + hypothermia 15 ± 2.4, one-way ANOVA, *p* < 0.01). Lactate levels in 12 and 12.5 min groups are similar to those observed in humans after cardiac arrest (Topjian et al., 2013).

### Injury in the Thalamic Reticular Nucleus After Cardiac Arrest

**FIGURE 2:**
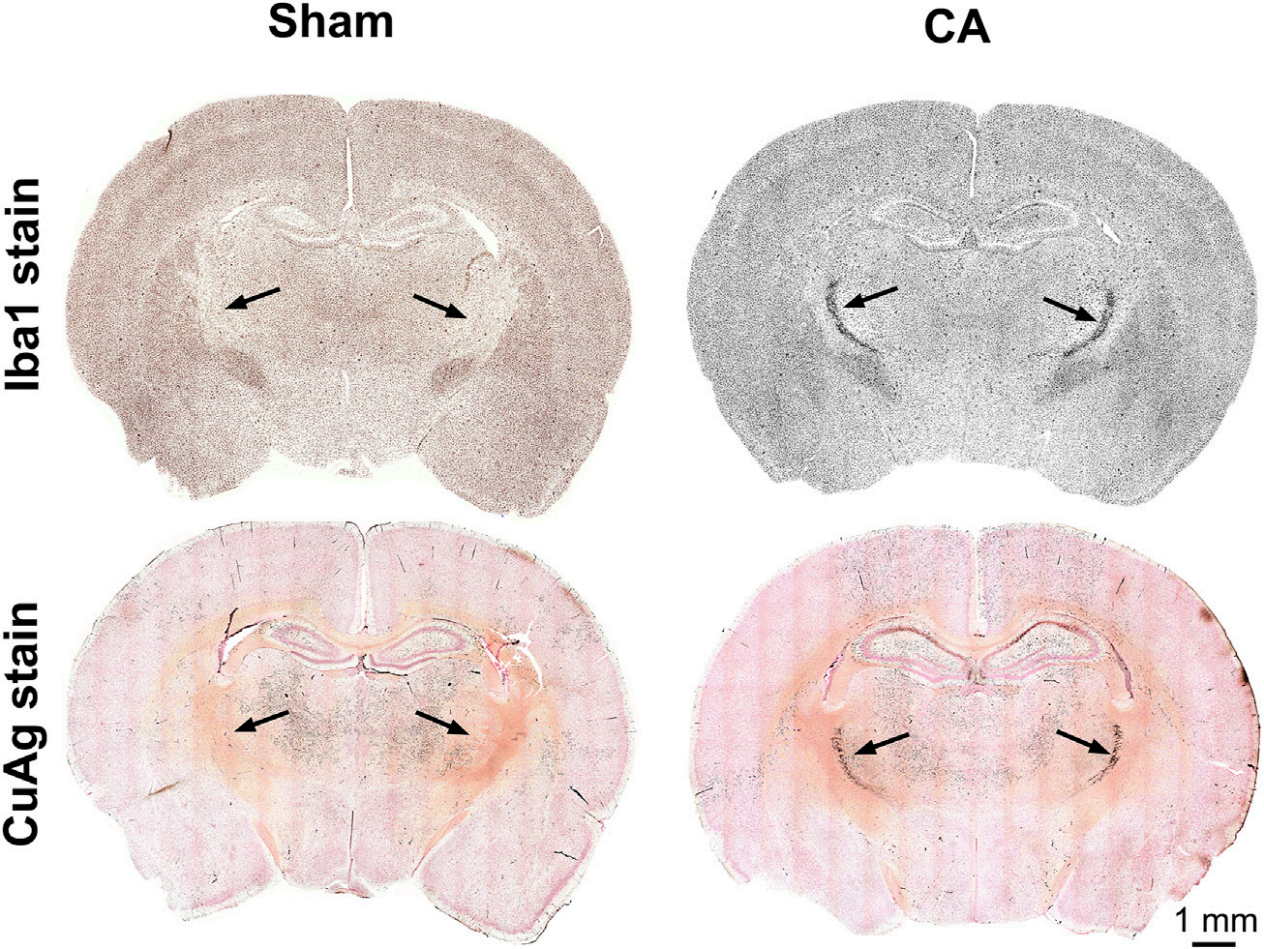
Coronal sections of CuAg and Iba1 labeling from sham and CA rats. The whole brain immunohistochemistry labelling Iba-1 and Amino Cupric Silver (CuAg) show the profound neuronal degeneration and microglial activation, respectively, in the specific region within the thalamus from 12.5 min CA compared to sham rats. The black arrows indicate the location of CA-induced injury in the thalamus, nRT.

**FIGURE 3:**
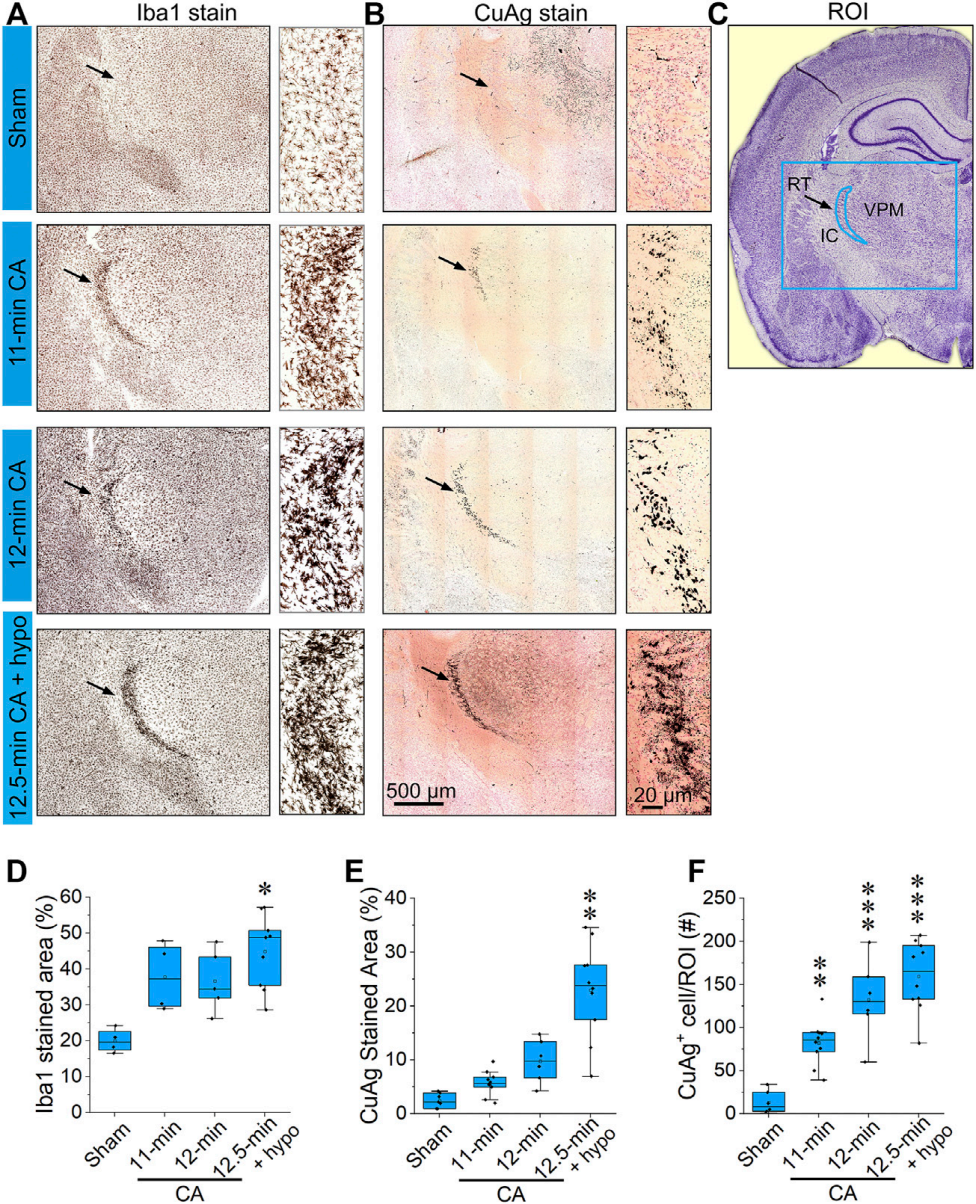
CA induces pronounced microglial activation and neurodegeneration in the thalamic reticular nucleus **(A,B)** Representative photomicrographs of Iba1 and Amino Cupric Silver (CuAg) stains in the reticular nucleus 24 h after 11; 12; 12.5 min CA and sham-operated rats **(C)** Schematic illustration of the reticular nucleus, the blue rectangle indicates the captured in A and B; the blue crescent-shape indicates the analyzed region. **(D–F)** The bar graphs show percentage of Iba1 stained area, CuAg-stained area, and the number of CuAg-stained cell in the ROI. It can be noted that both Iba1 and CuAg stains in RT from the CA rats show a remarkable increase compared to sham (Nested one-way ANOVA with Dunnett’s multiple comparisons test; ∗*p* < 0.05, ∗∗*p* < 0.01, ∗∗∗*p* < 0.001 vs sham group; Data are presented as individual values with median and interquartile range. Both left and right RT of 2,3 stained slides from each of 3–6 animals/group were analyzed). Scale bars represent 500 μm (low-power images) and 20 μm (high-power images).

The most prominent features of thalamic injury in this model of pediatric cardiac arrest and resuscitation are aggregation of activated microglia and neuronal degeneration in nRT 24 h after resuscitation (Figure 2). Severity and spatial extent of microglial activation and neuronal injury in nRT depend on cardiac arrest duration. Iba1 and CuAg staining increased in nRT of all rats that underwent cardiac arrest compared to sham-operated rats (Figure 3). The percent of nRT area covered by Iba1 staining increased as arrest duration increased (11 min CA: 37.83 ± 4.81%, *p* = 0.139; 12 min CA: 36.69 ± 3.87, *p* = 0.199; 12.5 min CA: 44.89 ± 3.39, *p* = 0.0188 vs sham: 19.98 ± 1.69%, Figure 3D). We did not attempt to quantify the number or the morphology of individual Iba1-positive cells in nRT due to near confluence of activated microglia in injured rats (Figures 2, Figure 3A). The percent of nRT area covered by CuAg staining also increased as arrest duration increased (11 min CA: 5.68 ± 0.72, *p* = 0.79; 12 min CA: 9.76 ± 1.64, *p* = 0.28; 12.5 min CA + hypothermia: 22.98 ± 2.77, *p* = 0.0012 vs sham: 2.46 ± 0.50%; Figure 3E). The morphology of CuAg staining demonstrates degeneration of neuronal somata and synaptic terminals in nRT. The number of degenerating nRT neurons identified by CuAg staining increased with arrest duration (11 min CA: 82.30 ± 8.22, *p* < 0.01; 12 min CA: 132.33 ± 19.03, *p* < 0.001; 12.5 min CA + hypothermia: 159.50 ± 12.96, *p* < 0.001 vs sham: 13.33 ± 5.05; Figure 3F, Nested one-way ANOVA with Dunnett’s multiple comparisons). Interestingly, both Iba1 and CuAg staining showed a continued increase in injury severity in the 12.5 min CA group despite use of mild hypothermia. These data suggest that nRT neurons are vulnerable to CA-associated hypoxic-ischemic injury early in the post-arrest recovery process. Furthermore, with arrest times in the rat model approaching those observed in children, mild hypothermia (34°C) does not prevent nRT injury.

### Spatial Gradients in nRT Injury After Cardiac Arrest

**FIGURE 4:**
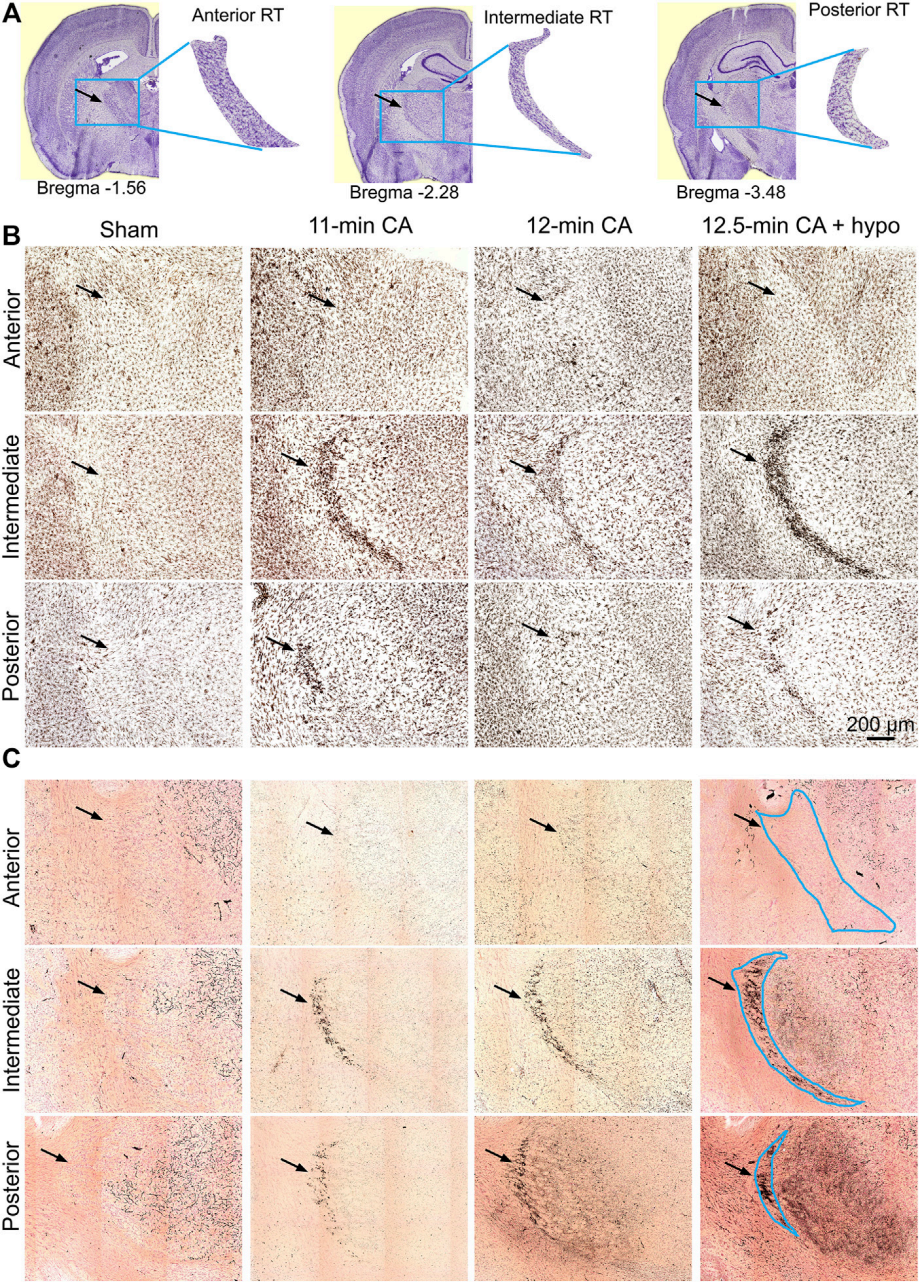
CA-induced microglia activation and neurodegeneration are observed in the posterior and intermediate but not in the anterior RT **(A)** Schematic identification of anteroposterior position and shape indicated with respect to bregma. The blue box (2000 μm × 1,600 μm) indicates the captured RT segments; the black arrowheads indicate the location of ROI **(B,C)** Composition of three Iba1-stained **(B)** and CuAg-stained **(C)** coronal sections through the anterior, intermediate and posterior of RT from each of the sham; 11 min, 12 min, and 12.5 min + hypothermia CA groups. Scale bar represents 200 µm applied for all images.

**FIGURE 5:**
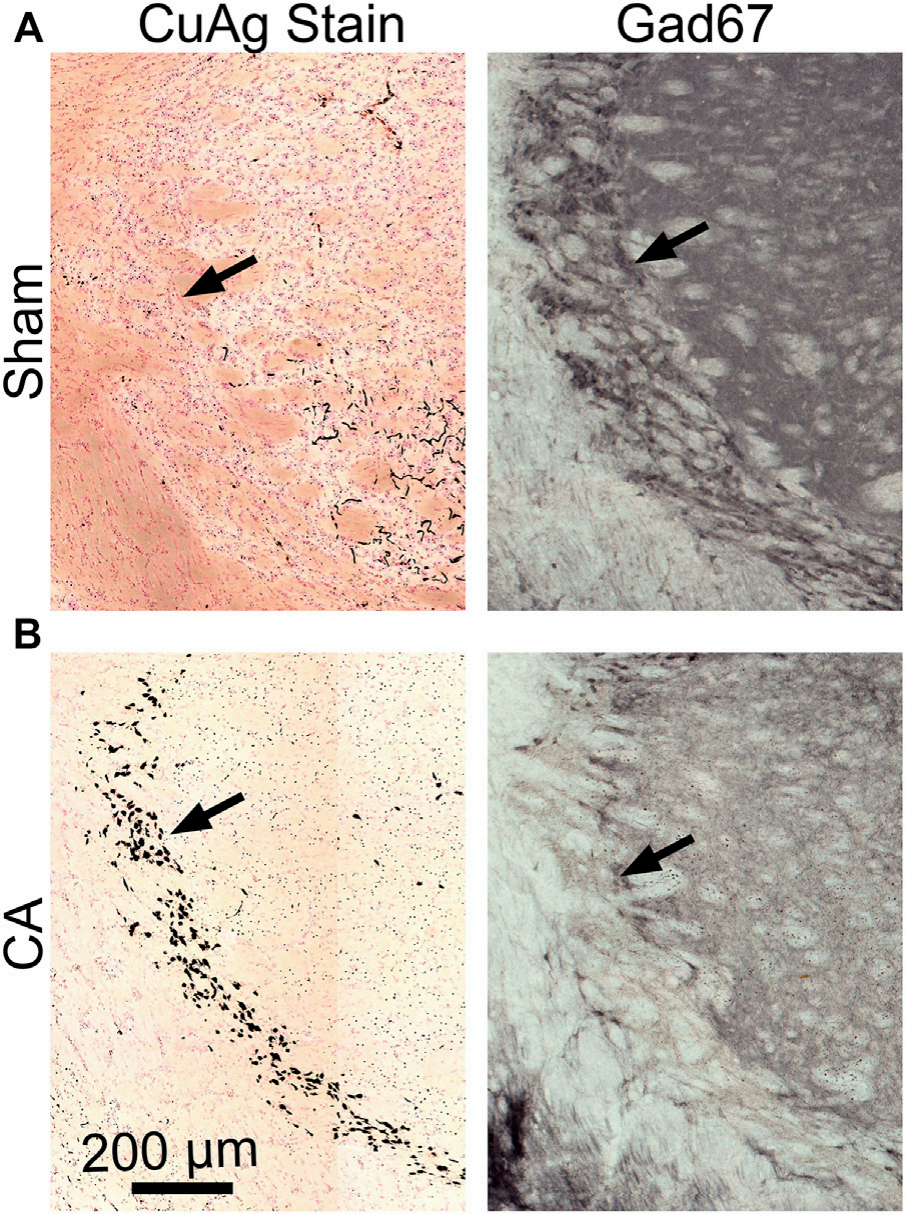
Gad67-stained and CuAg-stained sections from the same brain regions of the same representative sham or insult rats **(A)** The intermediate RT from sham section with lacking silver staining (left) shows prominent Gad67^+^ neurons while RT from CA section **(B)** with prominent CuAg^+^ stain in soma show a remarkable reduction of Gad67^+^ neurons. Scale bars represent 200 µM.

**FIGURE 6:**
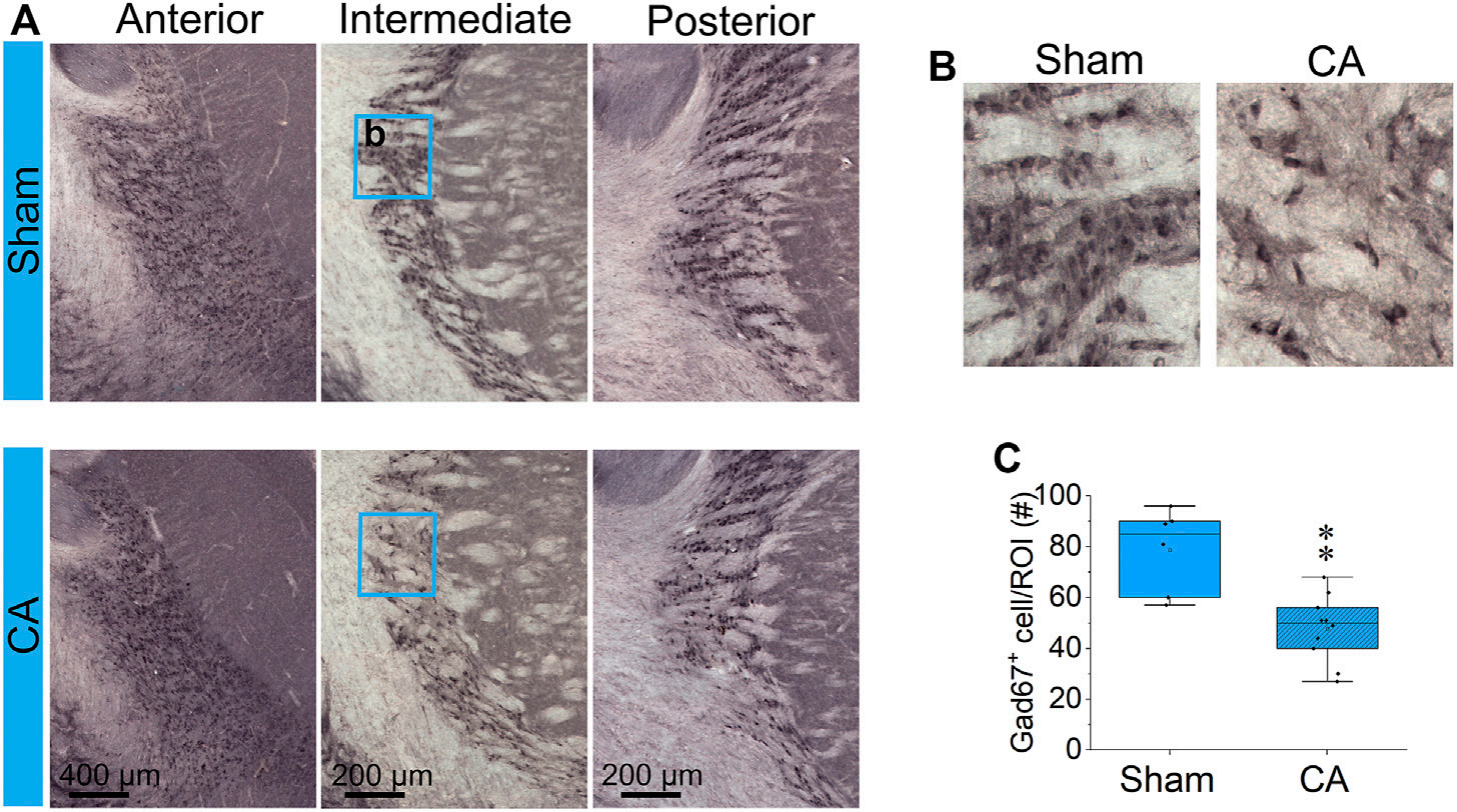
CA reduces GABAergic interneurons in the RT **(A)** Coronal Gad67-stained sections at anterior, intermediate and posterior RT from one representative brain in each of sham **(upper images)** and 12.5 min CA + hypothermia rats **(lower images)**. The blue rectangles (250 × 300 µm) capture the high magnification showed in **(B)** and analyzed Gad67^+^ cells in **(C)**. There is statistically significant decrease in the number Gad67-labeled neurons in intermediate RT between CA group compared with sham-operated group (Nested *t*-test, ∗∗*p* < 0.01). Data are presented as individual values with median and interquartile range from two to three stained slides from each of three sham and 5 CA animals.

**FIGURE 7:**
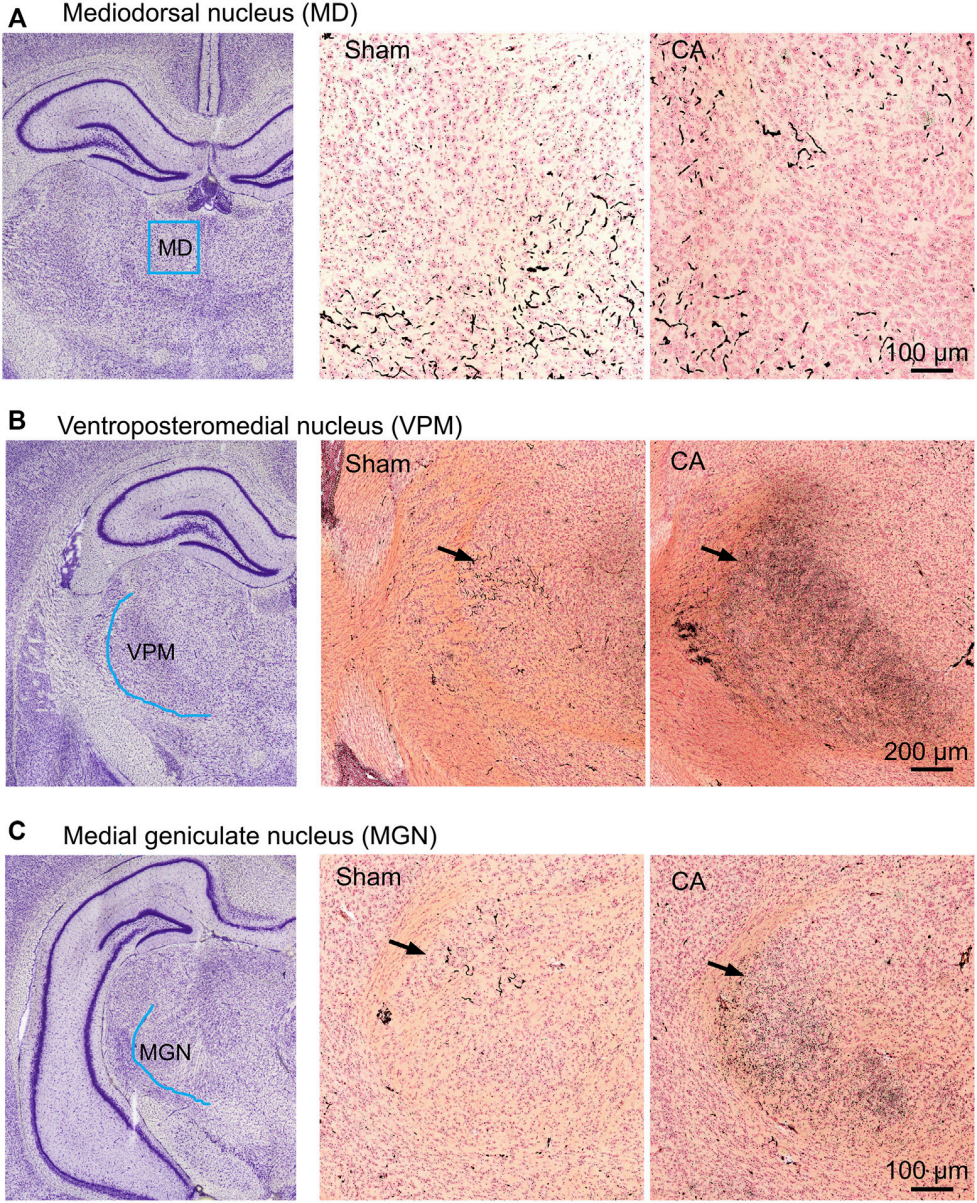
CA-induced synaptic degeneration in sub-areas of thalamic relays. The ROI scheme **(left)**, CuAg stain in sham **(middle)** and 12.5 min cardiac arrest **(right)** in the mediodorsal thalamic nucleus **(A)**, ventroposteriomedial **(B)**, and medial geniculate thalamic nucleus **(C)**. Note that the degenerating synapses were seen in both VPM and MGN but not in MD of injured animals.

The entire nRT in rodents comprises solely inhibitory GABA-ergic neurons (Jones, 2002). Anatomically, nRT is organized into anterior, intermediate and posterior segments. Functionally, nRT neurons in these segments process salient (anterior), somatosensory (intermediate) and auditory (posterior) information. Figure 4 shows CuAg- and Iba1-stained sections corresponding to each nRT segment (relative to bregma in *mm*, anterior -1.56, intermediate—2.28 and posterior—3.48). Neurodegeneration and microglial activation encompassed intermediate and posterior nRT segments while sparing anterior nRT. Using the most severely injured rats (12.5 min arrest + hypothermia), we quantified neuronal degeneration in intermediate nRT using Gad67 staining (Figures 5, 6). In individual rats, presence of CuAg staining correlated with absence of Gad67 staining (Figure 5). Even at this early stage after resuscitation, the number of Gad67^+^ neurons in intermediate nRT decreased in rats subjected to 12.5 min arrest despite application of hypothermia (Sham 79 ± 6.7, CA: 48 ± 4.1, *p* = 0.0012, Nested *t*-test; Figure 6). Finally, we evaluated degeneration in thalamic projection targets of nRT neurons. Anterior nRT projects to the mediodorsal thalamic nucleus (MD), intermediate–to the ventroposteriomedial thalamic nucleus (VPM), and posterior–to the medial geniculate nucleus (MGN). Consistent with degeneration patterns in the nRT, synaptic degeneration was observed in VPM and MGN but not in MD (Figure 7). These data indicate that neurons in intermediate somatosensory and posterior auditory nRT segments appear more vulnerable to CA-induced injury than neurons in the anterior salience segment.

### Lack of Degeneration Among Cortical GABA-Ergic Neurons After Cardiac Arrest

**FIGURE 8:**
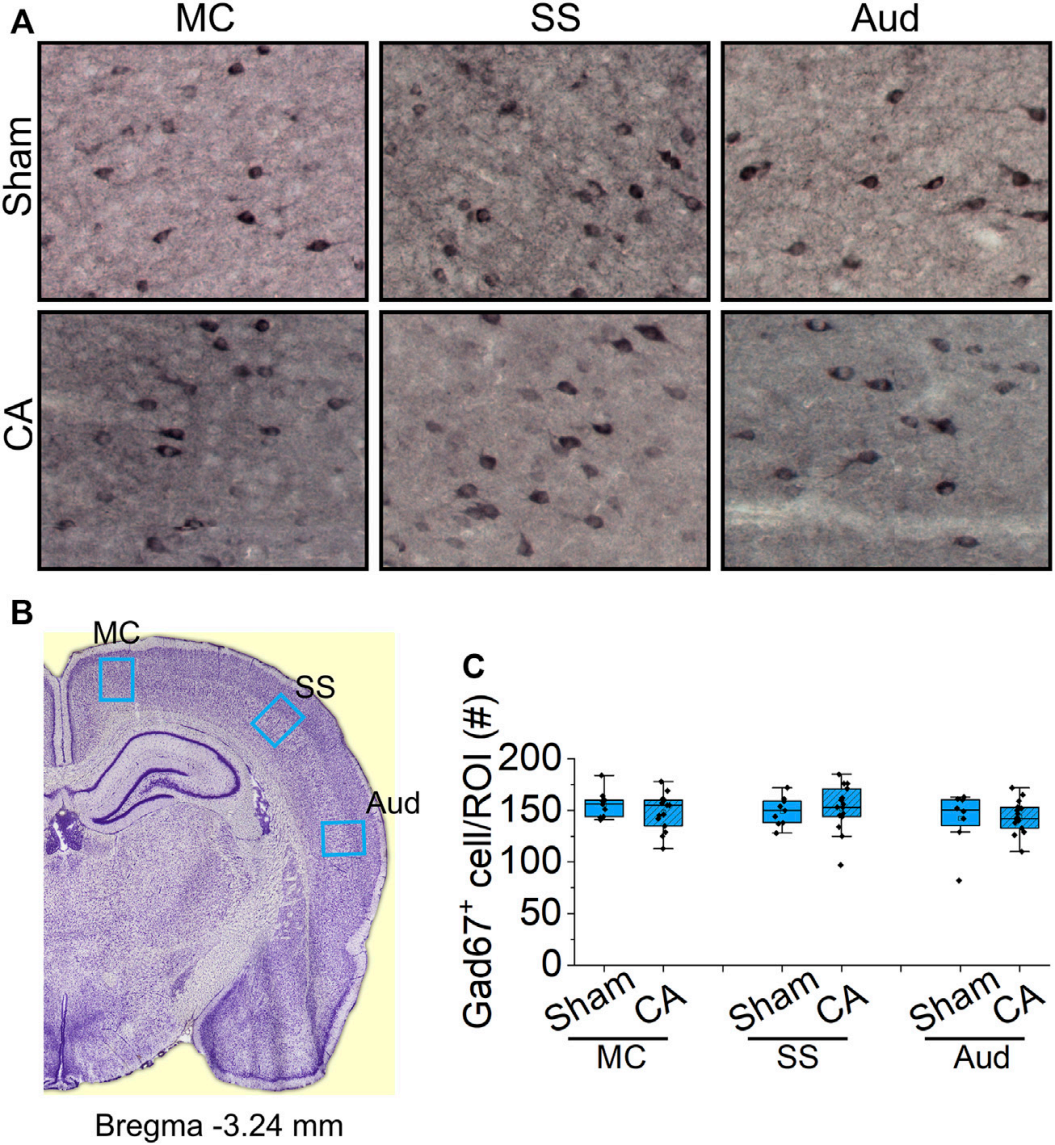
Analysis of GABA-ergic interneurons in the cortex of sham and CA **(A)** Representative images of Gad67 staining in the sub-regions of cortex from sham **(upper images)** and CA **(lower images)**. Blue arrows indicate the Gad67-labelled cells counted manually using multi-point function in FIJI **(B)** Coronal sections at bregma 3.24 mm with regions of interest marked by blue boxes: motor cortex (MC), somatosensory cortex (SS), and auditory cortex (Aud) **(C)** The graphs show the numbers of Gad67-stained cell with median and interquartile range in the ROIs from three stained slides from three sham and 5 12.5 min CA animals. There is no significant deference in the number Gad67-labeled neurons between CA group compared with sham-operated group (Nested *t*-test, *p* > 0.05).

The spatial gradient in cardiac arrest-induced degeneration among nRT neurons suggests that GABA-ergic identity is insufficient to explain their selective vulnerability to hypoxic-ischemic injury. We therefore investigated whether GABA-ergic cortical neurons also decrease in the most severely injured rats (12.5-min cardiac arrest + hypothermia). In three cortical areas examined–motor, somatosensory and auditory–the number of GAD67 + neurons remained unchanged 24 h after resuscitation (motor cortex: Sham 156 ± 4.47, CA 149 ± 4.53, *p* = 0.518; somatosensory cortex: Sham 149 ± 4.61, CA 152 ± 5.77, *p* = 0.793; and auditory cortex: Sham 142 ± 9.49, CA 144 ± 4.90, *p* = 0.746; Nested *t*-test; Figure 8). These data from the cerebral cortex, together with the lack of degeneration in the anterior nRT, suggest that selective vulnerability of intermediate and posterior nRT neurons to hypoxic-ischemic injury is unlikely to arise simply from their inhibitory identity.

## Discussion

We developed a model of severe pediatric asphyxial cardiac arrest, resuscitation and post-arrest intensive care in immature rats. The model approximates features of cardiac arrest in children, including arrest duration and post-arrest metabolic and physiologic disturbances. We then used this model to examine microglial activation and neuronal degeneration in the thalamus 24 h after resuscitation. Thalamic injury at this early time point is most prominent in the thalamic Reticular Nucleus. The injury is characterized by activation and aggregation of microglia and by degeneration of GABA-ergic neurons in the intermediate and posterior nRT segments. The injury is consistent, reproducible and titratable. We found that mild hypothermia fails to prevent neuronal degeneration and microglial activation in nRT at longer arrest durations. These anatomical data agree with the most recent clinical studies in children (Moler et al., 2015; Moler et al., 2017) and in adults (Nielsen et al., 2013) which suggest that compared to controlled normothermia, mild hypothermia does not improve cardiac arrest outcomes. Furthermore, we observed that the injury at this time is specific to a subset of nRT neurons, insofar as GABA-ergic neurons in the anterior nRT segment and in the cerebral cortex are spared at this early time point after arrest. These data identify a novel sub-population of GABA-ergic neurons that are selectively vulnerable to hypoxic-ischemic injury after cardiac arrest, suggest an interaction between these neurons and the surrounding microglia and provide potential targets for therapeutic intervention.

### Limitations

Our study has limitations. First, it examined only a single time point—24 h–after resuscitation. It is possible that other thalamic neurons degenerate at later times after arrest, similar to delayed neuronal death observed in the hippocampus 3–5 days after a milder hypoxic-ischemic injury (Pulsinelli et al., 1982; Horn and Schlote, 1992; Kirino, 2000). Second, the 12.5 min arrest + hypothermia group is compared to the 12 min arrest + normothermia (usual care) group. We found in preliminary experiments that rats subjected to 12.5 min arrest + normothermia had unacceptably high re-arrest rates after the initial resuscitation. We infer that injury would have been even more severe in that group. Third, we chose to forego the stereologic approach to counting cells (Gundersen, 1986; Peterson, 1999) because the histologic lesions were obvious and because volumetric estimates of cell density are not the objective of this study. Finally, we used rats in a single age group–PND17-19, and results may differ earlier or later in development.

### Selective Vulnerability

Inhibitory neurons as a population are generally thought to be vulnerable to hypoxic-ischemic injury during development. GABA-ergic cerebellar Purkinje cells degenerate after cardiac arrest in animal models (Paine et al., 2012; Au et al., 2015) and in humans (Hausmann et al., 2007). Similarly, Purkinje neurons show histologic and functional deficits in neonatal acute hypoxic-ischemic brain injury (Cervos-Navarro and Diemer, 1991) as well as in chronic hypoxia (Sathyanesan et al., 2018). Neonatal hypoxia-ischemia also results in loss of interneurons in the cerebral cortex (Fowke et al., 2018) and in the striatum (Galinsky et al., 2017). Yet, selective vulnerability may be a feature of specific sub-populations of GABA-ergic neurons rather than a general property of the entire population. Among Purkinje neurons, vulnerability to hypoxic-ischemic injury correlates with lack of expression of aldolase C and EAA4 glutamate transporter (Welsh et al., 2002). Our present data indicate the GABA-ergic neurons in the intermediate and posterior segments of the nRT are more vulnerable than those in the anterior segment. These findings support the hypothesis that GABA-ergic identity alone is not sufficient to explain selective vulnerability among inhibitory neurons.

Whence may selective vulnerability among nRT neurons arise? Differential perfusion of these segments as a cause can be eliminated outright, since all receive blood supply from the same perforating thalamic arteries. A recent report demonstrated presence of anterior-posterior gradients in gene expression in nRT neurons (Li et al., 2020). Gene expression gradients were associated with a physiologic gradient in firing properties of nRT neurons. It is possible that these gradients underlie selective vulnerability of intermediate and posterior nRT neurons or, conversely, resistance of anterior nRT neurons to injury after cardiac arrest. Additionally, selective vulnerability may arise from excess excitatory synaptic input onto defined populations of GABA-ergic neurons. For example, degeneration of Purkinje cerebellar neurons after hypoxia-ischemia requires ongoing excitatory input from the inferior olivary nucleus (Welsh et al., 2002). Neurons in intermediate and posterior nRT receive ongoing excitatory input from both sensory thalamocortical neurons in VPM and MGN, respectively, and from descending corticothalamic fibers. It is possible that post-arrest patterns of excitatory input onto the intermediate and posterior nRT neurons differ from those onto anterior nRT neurons, contributing to the observed differences in vulnerability to injury. Interestingly, several days after cardiac arrest and resuscitation, activity of VPM neurons is increased (Shoykhet et al., 2012). It is unknown, however, whether increased activity in VPM is a consequence of nRT injury and associated disinhibition, or if it actively contributes to neuronal degeneration.

### Functional Implications

Thalamic Reticular Nucleus provides the major source of inhibition to intrathalamic targets (Pinault, 2004). Topographic organization of intrathalamic nRT projections (Pinault and Deschenes, 1998) allows for exquisite inhibitory control of sensory information processing (Hartings et al., 2003; Fisher et al., 2017) and of higher order functions, such as sleep (Steriade, 1994) and attention (McAlonan et al., 2006; Wimmer et al., 2015). Recently, a first-in-humans study used functional MRI to confirm nRT’s involvement in vision and, surprisingly, to show that nRT participates in interhemispheric transfer of sensory information (Viviano and Schneider, 2015). Given the cardinal role of nRT in perception and cognition, injury to nRT neurons likely carries substantial morbidity. In animal models, anatomic or functional abnormalities in nRT exacerbate pain (Hornung et al., 2020), impair sensory perception (Shoykhet et al., 2012; Middleton et al., 2017), disrupt sleep (Fernandez et al., 2018) and attention (Wimmer et al., 2015; Wells et al., 2016). In humans, nRT dysfunction is associated with intractable pain (Gustin et al., 2014), autism (Chaudhry et al., 2015; Wells et al., 2016), schizophrenia (Ghoshal et al., 2020), and spike-and-wave seizures (Andy and Jurko, 1986; Crunelli et al., 2020). Perhaps relatedly, pain is common in cardiac arrest survivors (Boyce-van der Wal et al., 2015). Rhythmic spike-and-wave discharges are also frequent and portend a poor outcome in comatose patients after cardiac arrest (Westhall et al., 2016). In addition, burst suppression–another EEG rhythm likely driven by nRT and characterized by abnormal thalamocortical synchrony–occurs frequently in comatose cardiac arrest survivors (Westhall et al., 2016). Our findings, together with the wealth of clinical data, suggest that nRT injury likely contributes to severe neurologic deficits after cardiac arrest.

### Microglia-Neuron Interactions

Our data show pronounced accumulation of activated microglia in nRT after cardiac arrest. Currently, it is unknown whether microglial activation in nRT occurs simply in response to neuronal injury or whether it plays an additional pathologic role. In the hippocampus, microglial activation after cardiac arrest exacerbates neuronal degeneration (Chang et al., 2020). Ablation of microglia using minocycline ameliorates histologic injury (Tang et al., 2010), although impact may vary by cardiac arrest characteristics (Janata et al., 2019). In other brain hypoxia-ischemia models, microglial activation has both protective (Fleiss et al., 2021) and deleterious (Yew et al., 2019) effects. Interestingly, activated microglia generate nitric oxide (NO) among multiple other neurotoxic substances (Brown and Vilalta, 2015), and NO depolarizes primarily bursting nRT neurons in the thalamus (Yang and Cox, 2008). Thus, it is biologically plausible that microglial activation and aggregation in nRT contributes to early selective degeneration of nRT neurons after cardiac arrest. Our model of severe pediatric asphyxial cardiac arrest allows for testing this hypothesis *in vivo*.

## Conclusion

We developed a clinically relevant model of severe pediatric asphyxial cardiac arrest and resuscitation in immature rats. The model approximates physiologic disturbances observed in children after cardiac arrest, including the need for sustained intensive care after resuscitation. Using this model, we show early, selective degeneration of GABA-ergic neurons in the intermediate and posterior segments of the thalamic Reticular Nucleus. Neuronal degeneration occurs together with accumulation of activated microglia in nRT. Future studies need to determine the molecular basis of selective degeneration in a subset of nRT neurons, the contributing role of microglial activation in nRT and the behavioral consequences of nRT injury in cardiac arrest survivors. Finally, nRT injury in human cardiac arrest survivors remains to be characterized anatomically and functionally.

## Data Availability

The original contributions presented in the study are included in the article/Supplementary Material, further inquiries can be directed to the corresponding author.
