# Supplementary material for: Early Thalamic Injury After Resuscitation From Severe Asphyxial Cardiac Arrest in Developing Rats
Source: Front Cell Dev Biol. 2021 Dec 7;9:737319. doi: 10.3389/fcell.2021.737319 (PMC8688916; doi:10.3389/fcell.2021.737319)

NEUROSCIENCE ASSOCIATES, INC.
Premier Neurohistologic Services

Neurohistology Embedding, Sectioning & Staining

A total of 16 rat brains were received at NeuroScience Associates, Inc. (NSA). The brains were examined, then treated overnight with 20% glycerol and 2% dimethyl sulfoxide in phosphate buffered saline (PBS) to prevent freeze-artifacts. The specimens were then embedded, with up to 16 rat brains per block, arranged for coronal sectioning in a gelatin matrix using MultiBrain® Technology (NeuroScience Associates, Knoxville, TN), and arranged as shown in the Appendix.

After curing with a formaldehyde solution, the blocks were rapidly frozen by immersion in 2-methylbutane chilled with crushed dry ice and mounted on a freezing stage of an AO 860 microtome. The MultiBrain® blocks were sectioned coronally with a setting on the microtome of 40µm. All sections were cut through the entire brain less olfactory bulbs and collected sequentially into a series of 24 cups. All cups contained Antigen Preserve solution (50 parts PBS pH7.0, 50 parts ethylene glycol, 1 part polyvinyl pyrrolidone); no sections were discarded.

• Immunohistochemistry – For Iba1 and GFAP immunohistochemistry (IHC), every twenty-fourth section (at an interval of 960 microns), was stained free-floating. For GAD67 IHC every twelfth section (at an interval of 480 microns), was stained free-floating. All incubation solutions from the primary antibody onward used Tris buffered saline (TBS) with Triton X100 as the vehicle; all rinses were with TBS.

The Iba1 sections were rinsed in TBS then pretreated in sodium citrate buffer pH 6.0 in a water bath at 80ºC for antigen retrieval then rinsed. After a hydrogen peroxide treatment and rinses, the sections were immunostained with the primary antibodies as shown below, overnight at room temperature. Vehicle solutions contained Triton X100 for permeabilization. Following rinses, a biotinylated secondary antibody (anti IgG of host animal in which the primary antibody was produced) was applied. After further rinses Vector Lab's ABC solution Catalog # PK-6100 (avidin-biotin-HRP complex; details in instruction for VECTASTAIN® Elite ABC, Vector, Burlingame, CA) at a dilution of 1:222 was applied. The sections were again rinsed, then treated with a chromagen: diaminobenzidine tetrahydrochloride (DAB) and hydrogen peroxide to create a visible reaction product. The chromagen for the Iba1 and GAD67 stained sections included nickel (II) sulfate as noted below. Following further rinses, the sections were mounted on gelatin coated glass slides, then air dried. All stained slides were dehydrated in alcohols, cleared in xylene and coverslipped.

Primary Antibody:	Iba1 IHC
Source:	Wako
Catalog #:	019-19741
Host:	Rabbit
Dilution: 1:	12,000
Chromagen:	Ni(II) DAB
Stain Color:	Black
Secondary Antibody:	Anti-Rabbit Biotinylated
Source:	Vector
Catalog #:	BA-1000
Host:	Goat
Dilution: 1:	1,000

Primary Antibody: GFAP IHC
Source: Dako
Catalog #: Z0334
Host: Rabbit
Dilution: 1: 14,000
Chromagen: Ni(II) DAB
Stain Color: Brown
Secondary Antibody: Anti-Rabbit Biotinylated
Source: Vector
Catalog #: BA-1000
Host: Goat
Dilution: 1: 1,000

Primary Antibody: GAD67
Source: Abcam
Catalog #: ab26116
Host: Mouse
Dilution: 1: 30,000
Chromagen: Ni(II) DAB
Stain Color: Black
Secondary Antibody: Anti-Mouse Biotinylated
Source: Vector
Catalog #: BA-2001
Host: Horse
Dilution: 1: 1,000

• **Thionine Nissl Stain** - A set of every twenty fourth section (at an interval of 960 microns) was mounted on gelatin coated glass slides, air dried and carried through the following sequence: 95% ethanol, 95% ethanol/Formaldehyde; 95% ethanol, Chloroform/Ether/absolute ethanol (8:1:1), 95% ethanol; 10% HCl/ethanol, 95% ethanol, 70% ethanol, dH_2O, Thionine (0.05% Thionine/acetate buffer, pH 4.5), dH_2O, 70% ethanol, 95% ethanol, Acetic Acid/ethanol, 95% ethanol, 100% ethanol, 100% ethanol, 1:1 100% ethanol/xylene, xylene, xylene, coverslip.

• **Ischemia Contrast stain** – A set of every twelfth section (an interval of 480 microns) and a set of every twenty fourth section (at an interval of 960 microns) was mounted on gelatin coated glass slides, air dried then stained with a modification of the Weil method for myelin. The slides were dehydrated through alcohols, then rehydrated and stained in Hematoxylin/ Ferric Ammonium Sulfate staining solution. They were then differentiated first in 2% Ferric Ammonium Sulfate, rinsed in deionized water rinses, and secondly in a Potassium Ferricyanide/ Sodium Borate solution. The degree of differentiation in the second bleach is much less than that required for demonstrating myelin. Unlike myelin staining, the gray matter is left dark in order to highlight ischemic areas. Following deionized water rinses, the slides were dehydrated in a standard alcohol series, cleared in xylene and coverslipped.

• Amino Cupric Silver Stain - A set of every twelfth section (an interval of 480 microns) was stained free-floating using the de Olmos amino cupric silver (AmCuAg) method (de Olmos, JS et al; Neurotox. and Teratol. 16 545-561, 1994). The staining consisted of the following major steps: Pre-Impregnation, Impregnation, Reduction, Bleaching and Fixing.

The Pre-Impregnation solution contained: cupric nitrate, silver nitrate, cadmium nitrate, lanthanum nitrate, neutral red, alpha-amino butyric acid, alanine, pyridine, triethanolamine, isopropanol and deionized water (dH_2O). After the components were well mixed, the solution was microwaved until it reached 45-50°C. The solution was allowed to cool to room temperature, then filtered. The sections were rinsed in PBS then placed in buffered formaldehyde and subsequently rinsed with dH_2O. They were then placed into a dish containing the Pre-Impregnation solution in a ~47°C water bath. The dish was removed from the water bath, covered and allowed to cool overnight to room temperature away from light.

The Impregnation (silver diammine) solution contained silver nitrate, 100% ethanol, acetone, lithium hydroxide, ammonium hydroxide and dH_2O. The sections were removed from the Pre-Impregnation solution, rinsed with dH_2O, then acetone, and then placed into the Impregnation solution at room temperature.

The Reducer solution contained 100% ethanol, formaldehyde, citric acid and dH_2O. The sections were transferred from the Impregnation solution into the Reducer solution and placed in a water bath with a maintained temperature between 32-35°C. After the Reducer solution, the sections were transferred into dH_2O rinses.

The two Bleaching solutions contained potassium ferricyanide in potassium chlorate with lactic acid, and potassium permanganate with weak sulfuric acid. The sections were rapidly transferred through these bleaching solutions, fixed with sodium thiosulfate and then cleared in Kodak Rapid Fixer Solution. The sections were then rinsed in dH_2O after each solution above, and transferred to PBS. The sections were then mounted on gelatinized slides and were allowed to drain and air dry at room temperature. The dried slides were counterstained with Neutral Red to reveal normal cell bodies.

• Neutral Red Counterstain - The mounted slides were carried through the following sequence: 95% ethanol, 95% ethanol/formaldehyde; 95% ethanol, 70% ethanol, dH_2O, neutral red solution made in an acetate buffer; pH4.5, dH_2O, differentiated in 70% ethanol, 95% ethanol, 100% ethanol, 1:1 100% ethanol/xylene, xylene, then coverslipped with Permount as a bonding medium.

Slide identification:
Each slide was laser etched with the block number and the stain. Following serial ordering of the slides, rostral to caudal for each stain, the slides were numbered by permanent ink in the upper right corner.

Appendix

Arrangement of brains for MultiBrain® embedding and the appearance on the finished slides of the sections from the individual brains. Dot in the lower right corner identifies left and right. Additional dots assist with block identity.

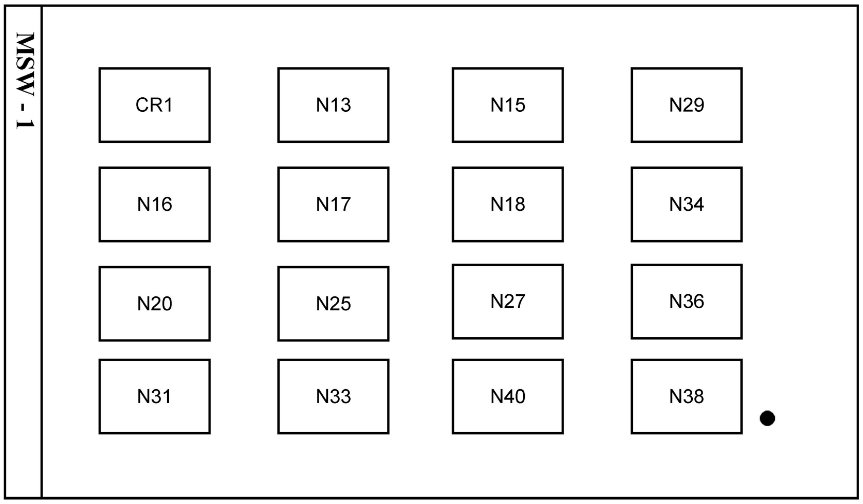

Supplement: Supplementary file 1 [file DataSheet1.PDF]
